# The Impact of miRNA Target Sites in Coding Sequences and in 3′UTRs

**DOI:** 10.1371/journal.pone.0018067

**Published:** 2011-03-22

**Authors:** Zhuo Fang, Nikolaus Rajewsky

**Affiliations:** Max Delbrück Centrum für Molekulare Medizin, Berlin, Germany; Virginia Commonwealth University, United States of America

## Abstract

Animal miRNAs are a large class of small regulatory RNAs that are known to directly and negatively regulate the expression of a large fraction of all protein encoding genes. The identification and characterization of miRNA targets is thus a fundamental problem in biology. miRNAs regulate target genes by binding to 3′ untranslated regions (3′UTRs) of target mRNAs, and multiple binding sites for the same miRNA in 3′UTRs can strongly enhance the degree of regulation. Recent experiments have demonstrated that a large fraction of miRNA binding sites reside in coding sequences. Overall, miRNA binding sites in coding regions were shown to mediate smaller regulation than 3′UTR binding. However, possible interactions between target sites in coding sequences and 3′UTRs have not been studied. Using transcriptomics and proteomics data of ten miRNA mis-expression experiments as well as transcriptome-wide experimentally identified miRNA target sites, we found that mRNA and protein expression of genes containing target sites both in coding regions and 3′UTRs were in general mildly but significantly more regulated than those containing target sites in 3′UTRs only. These effects were stronger for conserved target sites of length 7–8 nt in coding regions compared to non-conserved sites. Combined with our other finding that miRNA target sites in coding regions are under negative selection, our results shed light on the functional importance of miRNA targeting in coding regions.

## Introduction

MicroRNAs are important trans-acting genes that post-transcriptionally regulate the expression of a large number of protein-coding genes in metazoans [1]–[4]. In animals, miRNAs are thought to regulate gene expression by mediating repression of protein synthesis and/or destabilization of mRNAs [5], although recent studies have indicated that in the majority of reported cases, a reduction in protein synthesis can be explained by direct down-regulation of target mRNA levels [6], [7]. It is now widely accepted that a primary determinant for miRNA binding is usually perfect, consecutive Watson-Crick base-pairing between the target mRNA and the miRNA at position 2–7 in the 5′ end of the mature miRNA [8]. These hexamers in target mRNAs are also called seed sites or seeds and are believed to nucleate the binding event [8], [9]. Functional seeds are generally located in the 3′UTRs of mRNAs. Numerous studies have shown that multiple seed sites in the same 3′UTR confer much stronger regulation than single seed sites. However, reporter assay experiments have suggested that miRNA targeting can also occur in coding regions [10], [11]. These reporter assays are supported by biochemistry based methods that allow the genome-wide identification of mRNA binding sites of the miRNA effecter complex RISC [12]. Furthermore, large-scale miRNA mis-expression studies also have suggested that seeds in coding regions can confer regulation but are on average less effective than those in 3′UTRs [6], [7]. However, these studies analyzed seeds in coding regions independent of the existence of seeds in 3′UTRs. Only one study examined possible synergistic effects of seeds occurring both in coding regions and 3′UTRs but the experiment was done on only one reporter construct [11].

To further understand the contribution of miRNA seeds in coding regions and their possible interaction with 3′UTR seeds, we analyzed the composite effect of miRNA seeds in coding regions and 3′UTRs by using large-scale, genome wide miRNA mis-expression data that were published recently by two laboratories [6], [7]. In our own study, miRNAs were over-expressed and knocked down in cell lines (HeLa), and changes in the number of newly synthesized proteins, integrated over 12 hours, were quantified together with changes in mRNA levels for thousands of genes [6]. In the other study, the response in protein (but not in protein synthesis) and mRNA levels were measured after adding miRNAs into cultured cells and deleting miR-223 in mouse neutrophils [7]. In both studies, conservative estimates showed that down/up-regulated genes were enriched in direct, seed mediated miRNA targets [6], [7]. We noticed that changes in protein synthesis [6] seem generally stronger than changes in protein levels [7] (NR and ZF, unpublished results). Nevertheless, since we are interested in the general strength of regulation mediated by seed sites, we analyzed both data sets together.

## Results

### miRNA target sites in coding regions and 3′UTRs have synergistic effects

We re-analyzed data from altogether ten experiments published by two laboratories in [6] and [7]. The over-expression experiments were performed with miR-1, miR-30a, miR-155, miR-16, let-7b, miR-124, miR-181, among which the miR-1 over-expression were carried out by both studies [6], [7]. The two knockdown experiments were performed with let-7b in Hela cells [6] and miR-223 in mouse neutrophils [7]. We applied our analyses separately on log2 fold changes of mRNA and protein levels after miRNA mis-expression compared to control mRNA and protein levels. For the knockdown experiments, the sign of log2 fold changes were flipped so that they can be treated jointly with the over-expression experiments. In what follows, we grouped proteins by certain features of their mRNA sequences. Four groups of mRNAs/proteins were compared. These groups were 1) mRNAs/proteins that have no seed in 3′UTRs or coding regions; 2) mRNAs/proteins that have seeds only in coding regions; 3) mRNAs/proteins that have one seed in 3′UTRs and no seed in coding regions; 4) mRNAs/proteins that have one seed in 3′UTRs and additional seeds in coding regions.

**Figure 1:**
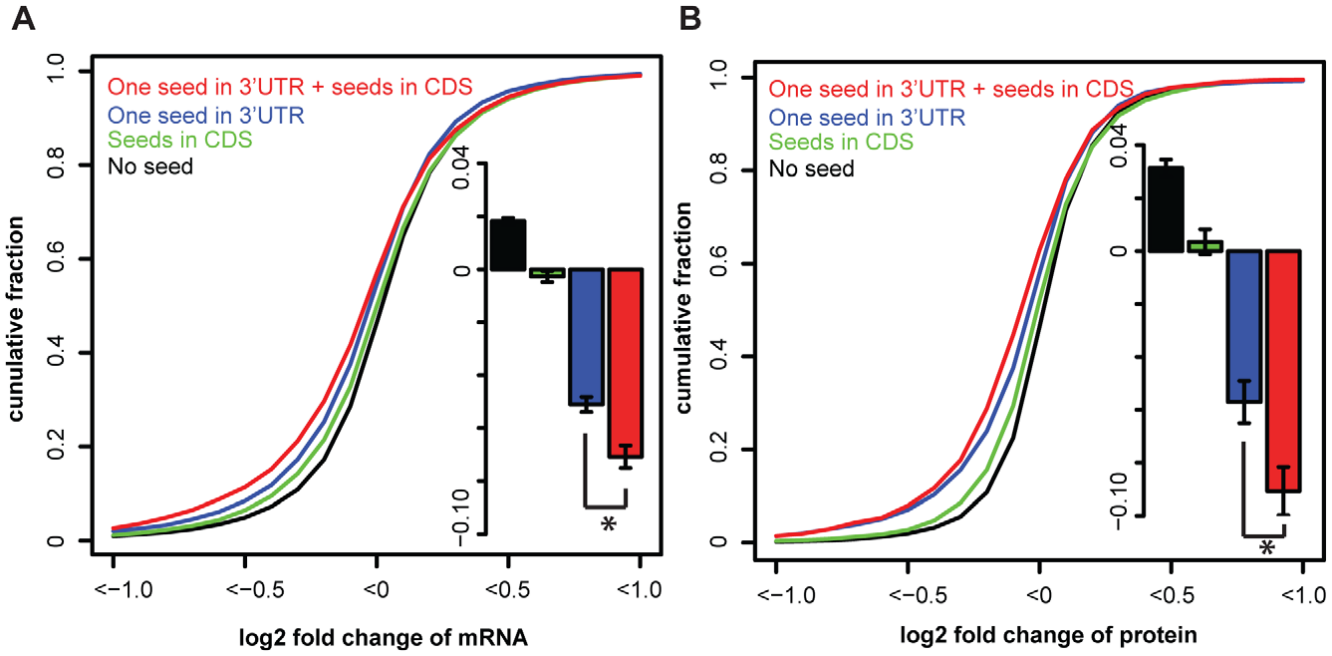
miRNA target sites in coding regions act synergistically with seeds in 3′UTRs. A) Cumulative distributions of log2 fold changes of mRNAs containing one miRNA seed in 3′UTRs and additional seeds in coding regions, one seed in 3′UTRs, seeds only in coding regions and no seed, respectively. The insert shows the mean log2 fold changes (with standard error) of corresponding mRNA groups. *P-value <10^−5^ by Wilcoxon test and <10^−11^ by Kolmogorov-Smirnov test. B) The same as A) for protein changes. *P-value <10^−3^ by Wilcoxon test and <10^−5^ by Kolmogorov-Smirnov test. Results are shown for pooled data of 10 miRNA mis-expression experiments.

Genes without any seed were slightly up-regulated (average log2 mRNA fold change is 0.018 and average log2 protein fold change is 0.031, Figure 1 A and B). They were used as background model of gene expression. The up-regulation of background genes may be due to the secondary effects of miRNA mis-expression or normalization issues. Genes containing miRNA seeds in coding regions but no seed in 3′UTRs were slightly down-regulated (average log2 mRNA fold change −0.003, average log2 protein fold change 0.004, Figure 1 A and B) compared to background (p-value <10^−15^ both for mRNA and protein, one-sided Wilcoxon test) but had smaller fold changes (p-value <10^−15^ both for mRNA and protein, one-sided Wilcoxon test) than genes with one seed in 3′UTRs (average log2 mRNA fold change is −0.051, average log2 protein fold change is −0.057, Figure 1 A and B). This is consistent with previous results in [6], [7]. However, mRNAs with the same number of 3′UTR seeds (one seed) and with additional seeds in coding regions (average log2 mRNA fold change is −0.071, average log2 protein fold change is −0.091, Figure 1 A and B) were more regulated than genes without seed in coding regions (P-value <10^−15^ for mRNA and P-value <10^−3^ for protein, one-sided Wilcoxon test), indicating that miRNA seeds in coding regions can, overall, mildly but significantly enhance regulatory effects mediated by 3′UTR seeds. This effect was consistent for most of the experiments (8 out of 10) when examining each of them individually. We will refer to this effect as ‘synergistic’. Detailed information about the different gene groups is given in Table S1 and Table S2.

The analysis so far only considered computationally defined miRNA target sites, and thus may not reflect context dependent gene regulation by miRNAs. We therefore repeated the same analysis on transcriptome-wide experimentally identified miRNA binding sites in a human cell line [12] combined with miR-124 over expression data in the same cell line [13] (Methods). The synergistic effect of miRNA target sites in coding regions with sites in 3′UTRs was consistent with what we described above (Figure S1).

### miRNA target sites in coding regions are under negative selection

Sequences that are conserved among species are in general more likely to be functional than non-conserved ones. It is well known that miRNA targets with conserved seeds in 3′UTRs confer significantly more regulation than the ones with non-conserved seeds, both on mRNA and protein levels [6], [7]. Not surprisingly, miRNA target prediction algorithms using seed conservation as a search criteria have more accuracy than the others [6], [7]. Similarly, it has been reported that after let-7 over expression, mRNAs with conserved let-7 seeds in coding regions were more down-regulated than mRNAs with non-conserved seeds [15]. We asked if it is possible to computationally detect negative selection on miRNA target sites in coding sequences. To answer this question, we used computational methods which have been well established for miRNA target sites in 3′ UTRs [1], [2]. The idea is to generate artificial miRNA sequences and to count the number of instances in which the corresponding seed sites appear to be conserved. This number represents background conservation of seed sites (“noise”) and is compared to the “signal”, i.e. the number of instances in which seed sites of real miRNAs are conserved. For this method, it is important that the total number of seed sites (conserved and not conserved) for the artificial miRNAs is comparable.

**Figure 2:**
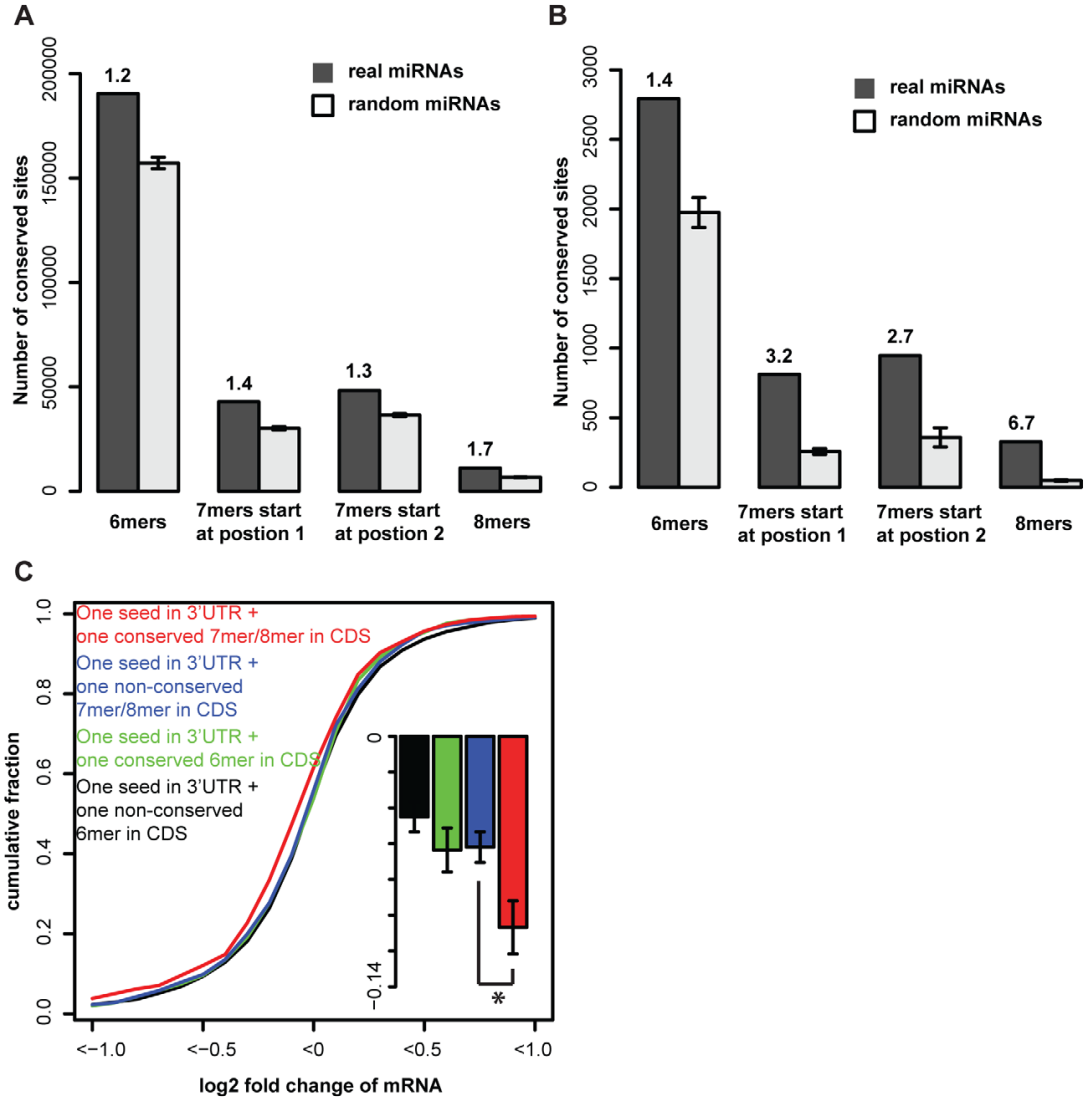
Conserved 7−8 nt miRNA target sites in coding regions mediate more synergistic effects than 6 nt sites. A) Signal-to-noise ratios for miRNA seeds conservation among 5 species in coding region. Bars indicate the number of conserved sites for 6mers, 7mers starting at position 1, 7mers staring at position 2, and 8mers for real miRNAs and random miRNAs. Standard errors are computed across six random miRNAs per real miRNA. Signal-to-noise ratios are represented above the bars. B) The same for 11 species. C) Cumulative distributions of log2 fold changes of mRNAs containing one miRNA seed in 3′UTRs and an additional conserved 7/8mer in coding regions, non-conserved 7/8mer, conserved 6mer and non-conserved 6mer, respectively. The insert shows the mean log2 fold changes (with standard errors) of corresponding mRNA groups. *P-value <10^−3^ by Wilcoxon test and <10^−4^ by Kolmogorov-Smirnov test.

Briefly, we selected 45 miRNAs which represent unique human miRNA families and conserved in vertebrates (Table S3) and counted the number of conserved seeds of them in coding regions across 5 species and 11 species (Methods). The background model was generated by generating, individually for each of the 45 miRNAs, six groups of random sequences with equal length to miRNA seeds and the same distribution of miRNA seed occurrences in human coding sequences (Methods). Signal-to-noise ratios were computed for 6mer seeds (matches to position 2–7 of 5′ miRNA sequences), 7mer seeds starting at position 1 (matches to position 1–7), 7mer seeds starting at position 2 (matches to position 2–8), 8mer seeds (matches to position 1–8) respectively, both in 5 species and 11 species alignments (ranging from human to dog/ tetraodon, respectively). Figure 2 A and B both show that 7mer and 8mer seeds have larger signal-to-noise ratios than 6mer seeds and 6mer seeds are only slightly better than random 6mers (signal-to-noise ratios are close to 1), suggesting that in coding region, conserved 7mer and 8mer seeds are more functional than conserved 6mer seeds.

### Conserved, extended miRNA target sites have more synergistic effect than short seeds

The negative selection of miRNA binding sites in coding regions was further supported by examining the mRNA expression profiles after miRNA misexpression. For mRNAs containing one 3′UTR seed, the ones which had an additional conserved 7mer/8mer seed in coding regions were in general more regulated than the ones have an additional non-conserved 7mer/8mer seed (average fold changes a−0.107 and −0.062, respectively; p-value <10^−3^, one-sided Wilcoxon test, Figure 2 C) while there was no significant difference between mRNAs with one additional conserved 6mer seed or non-conserved 6mer seed in coding regions (average fold changes −0.064 and −0.045, respectively; p-value 0.2, one-sided Wilcoxon test, Figure 2 C). Moreover, among additional conserved seeds in coding region, 7mer/8mer seeds confer more regulation than 6mer seeds (average fold changes −0.107 and −0.064, respectively; p-value <0.002, one-sided Wilcoxon test, Figure 2 C) but the same difference is not significant for non-conserved seeds (Figure 2 C). Thus, additional conserved 7mer/8mer seeds in coding region have more synergistic effect than conserved 6mer seeds on regulation of mRNAs.

## Discussion

Recent studies have demonstrated that miRNAs bind extensively to coding sequences [12] and that miRNA seeds in coding regions are correlated with regulation of targets [6], [7], [11]. Furthermore, reporter assay experiments confirmed that miRNAs seeds in coding regions can be functional [10], [11]. Nevertheless, the general opinion so far seems to be that seeds in coding regions have much smaller effect than seeds in 3′UTRs. Altogether, the function of seeds in coding regions is not understood. Our results show that miRNA target sites in coding regions enhance regulation mediated by target sites in 3′UTRs. This suggests that one possible function of seeds in coding regions is in helping recognition of seeds in 3′UTRs or in enhancing miRNA-target interactions. We note that this synergistic effect of seeds in coding sequences is significant but weaker than the effect generated by additional 3′UTR seeds. For example, for mRNAs containing two miRNA seeds, the ones which have one seed in 3′UTRs and one seed in coding regions were less regulated than those which have two 3′UTRs seeds (averaged log2 fold changes in above 10 experiments are −0.06 and −0.09, respectively; p-value < 0.01, one-sided Wilcoxon test).

Some sequence features in 3′UTRs have been reported to be related to the regulation level by miRNAs. For example, on average, extended seed matches are associated with greater regulation than shorter matches and conserved seeds exert more regulation than non-conserved seeds [6], [14], [16]. One problem in the interpretation of our results is that the detected enhancement of 3′UTR mediated regulation by seeds in coding region is just a correlation and therefore potentially the by-product of much more specific relationships between miRNA binding sites in coding regions and other known or unknown 3′UTR features. For example, it could be that miRNA target sites in coding sequences occur generally in mRNA with extended or conserved 3′UTR seeds. Although it is impossible to rule out such effects in general, we compared the occurrence of coding seeds for mRNAs with different length and conservation features of 3′UTR seeds. The results showed that the mRNAs containing coding seeds distributed almost uniformly in different groups and were not enriched in groups of mRNAs which have extended or conserved seeds in 3′UTRs (Table S4).

Previous studies showed that the more seeds mRNAs/proteins have in their 3′UTRs, the more strongly regulation is induced by miRNAs [6], [7], [14], [17]. On average, the log2 fold change correlated linearly with the seed number, which suggests that the effect of seeds is independent and multiplicative [6]. However, the correlation between seed number in coding regions and regulation is different. In some experiments (for example, miR-16 and let-7b over-expression), the synergistic effect is stronger when there are more seeds in coding region but it is marginal for the rest. Since the sequence length of coding regions varies largely between individual mRNAs, the absolute seed number in coding regions is biased towards mRNAs with long coding sequences. We therefore normalized the seed number in coding regions by dividing the total number of seeds in coding regions by the length of the coding sequence but still were not able to find any clear correlation between normalized seed numbers and regulation. Therefore, it seems that it is the presence, but not the number, of seed sites in coding regions that gives rise to the synergistic effect.

When examining whether miRNA seeds in different locations of coding region have different synergistic effect, we found that seeds residing very close to the stop codon frequently mediate very strong effects (two times more than the average effect) on mRNA levels in most experiments (eight/ten) (Figure S2). This strong signal was only observed in the region within ∼50 nt upstream of the stop codon. Since 3′UTR annotation is far from perfect, there is a possibility that these sites are in fact not truly in coding regions but in3′UTRs. However, manual inspection of the annotation for numerous sites by checking the publicly available evidence at the UCSC database (http://genome.ucsc.edu) did not support mis-annotation.

An interesting possibility is that the position of functional miRNA target sites within coding regions is correlated with splicing signals. We carefully checked the correlation between mRNA/protein changes with distances between miRNA seeds within coding regions and exon-exon juctions/splicing factor motifs. However, we did not find any signal (data not shown).

As shown in Figure 2 C, the synergistic effect for mRNA changes of conserved seeds in coding region is different for different length of base pairing. However, this difference was not visible for protein changes (P-value  = 0.677). We believe that this may be due to technical limitations in the experimental assays. As shown in [6], [7], state-of-the-art mass spectrometry can only quantify up to ∼5000 proteins and has a tendency to assay relatively highly expressed proteins.

Based on (a) experimental data clearly showing a large degree of miRNA binding in coding regions (b) our finding that seed sites in coding sequences are under negative selection (c) our finding that miRNA target sites in coding sequences can enhance regulation mediated by sites in 3′ UTRs, it seems clear that miRNA target sites in coding sequences are functionally important. We hope that this study will help future attempts to unravel their function.

## Methods

Microarray data and pSILAC data from [6] were downloaded from https://psilac.mdc-berlin.de. Microarray data at 32 h after transfection were used. Only “present” probes in microarrays were included. The “present” and “absent” information of microarray probes were obtained by applying the mas5calls() function in biocondutor (http://www.bioconductor.org). Protein identifiers were mapped to Refseq identifiers and sequence features were assigned with the same way in [6]. For the proteins which had more than one mapped Refseq identifiers, we randomly picked one identifier.

Microarray data and SILAC data from [7] were downloaded from http://www.nature.com/nature/journal/v455/n7209/suppinfo/nature07242.html. Data with Refseq identifiers were used without any additional filtering.

The 3′UTR and coding sequences were extracted from UCSC Genome Brower (http://genome.ucsc.edu, based on NCBI Build 36.1). Multiple, genome wide MultiZ alignments of 46 species to human genome (hg19/GRCh37, Feb. 2009) were also downloaded from UCSC Genome Brower (http://hgdownload.cse.ucsc.edu/goldenPath/hg19/multiz46way/).

The transcriptome-wide miR-124 binding sites were identified by a PAR-CLIP (Photoactivatable-Ribonucleoside-Enhanced Crosslinking and Immunoprecipitation) experiment in [12]. PAR-CLIP experiment was performed on miR-124-overexpressed FLAG/HA-AGO2 cells [12]. Crosslinking sites which have miR-124 seeds up-/down-stream within 20nt were taken into account. Corresponding miR-124 overexpression dataset was obtained from [13].

The conservation of real and random miRNA seeds in coding region among 5 species was computed by using human, chimpanzee, mouse, rat and dog. The conservation among 11 species were computed by using human, chimpanzee, mouse, rat, dog, elephant, armadillo, opossum, chicken, X.tropicalis and tetraodon. Only perfect and aligned matches in all species were considered.

Conserved miRNAs were selected by using a similar approach as in [2]. Mature and precursor miRNA sequences were downloaded from miRbase (release 14, http://www.mirbase.org/). Conserved miRNAs were selected from precursor alignments between human, chimpanzee, mouse, rat, dog, opossum, chicken, X.tropicalis and tetraodon. A miRNA was considered as conserved if most of positions in the mature and star sequences were conserved. 45 miRNAs were obtained by merging miRNAs that have identical seed sequences.

Random sequences were selected with equal length and approximately the same abundances (±15%) with real miRNA 6mer, 7mer and 8mer seeds in human coding sequences. Considering the particular feature of coding sequences, we additionally required that the random x-mers should have the same distribution of frame positions (±20%) with real miRNA x-mer seeds. Signal-to-noise ratios were computed as the ratios between number of conserved hits of real miRNA seeds and mean number of conserved hits for six groups of random miRNAs.

## Supporting Information

Figure S1 Effect of miRNA target sites in coding regions in AGO2 PAR-CLIP experiment. The figure shows cumulative distributions of log2 fold changes of mRNAs containing one AGO2 binding site in 3′UTRs and additional binding sites in coding regions, one binding site in 3′UTRs, binding sites only in coding regions and no binding site, respectively. The insert shows the mean log2 fold changes (with standard error) of corresponding mRNA groups. *P-value is 0.03 by Wilcoxon test and 0.01 by Kolmogorov-Smirnov test.
(TIF)

Figure S2 Strong synergistic effect of miRNA seeds in coding region within ∼50 nt of stop codon. mRNAs with 1 3′UTR seed and 1 seed in coding region were grouped according to the distances of the seed in coding region to the stop codon. Averaged log2 fold changes with standard errors were shown.
(TIF)

Table S1 Detailed information of mRNA/protein groups.
(XLS)

Table S2 Log2 fold changes of mRNAs/proteins with 1 3′UTR seed but no coding seed and mRNAs/proteins with 1 3′UTR seed and coding seeds. The identifiers of proteins were mapped to corresponding Refseq identifiers.
(XLS)

Table S3 45 unique human miRNAs which are conserved in human, chimpanzee, mouse, rat, dog, opossum, chicken, X.tropicalis and tetraodon.
(XLS)

Table S4 Fraction of mRNAs containing miRNA seeds in coding regions for mRNAs grouped by different 3′UTR sequence features. 1) 7mer means paring for position 2–8 or position 1–7 of miRNA sequences. 2) 8mer means paring for position 1–8 of miRNA sequences. 3) only expressed mRNAs in each experiment were considered.
(XLS)
