# Supplementary figures and images for: The Impact of miRNA Target Sites in Coding Sequences and in 3′UTRs
Source: PLoS One. 2011 Mar 22;6(3):e18067. doi: 10.1371/journal.pone.0018067 (PMC3062573; doi:10.1371/journal.pone.0018067)

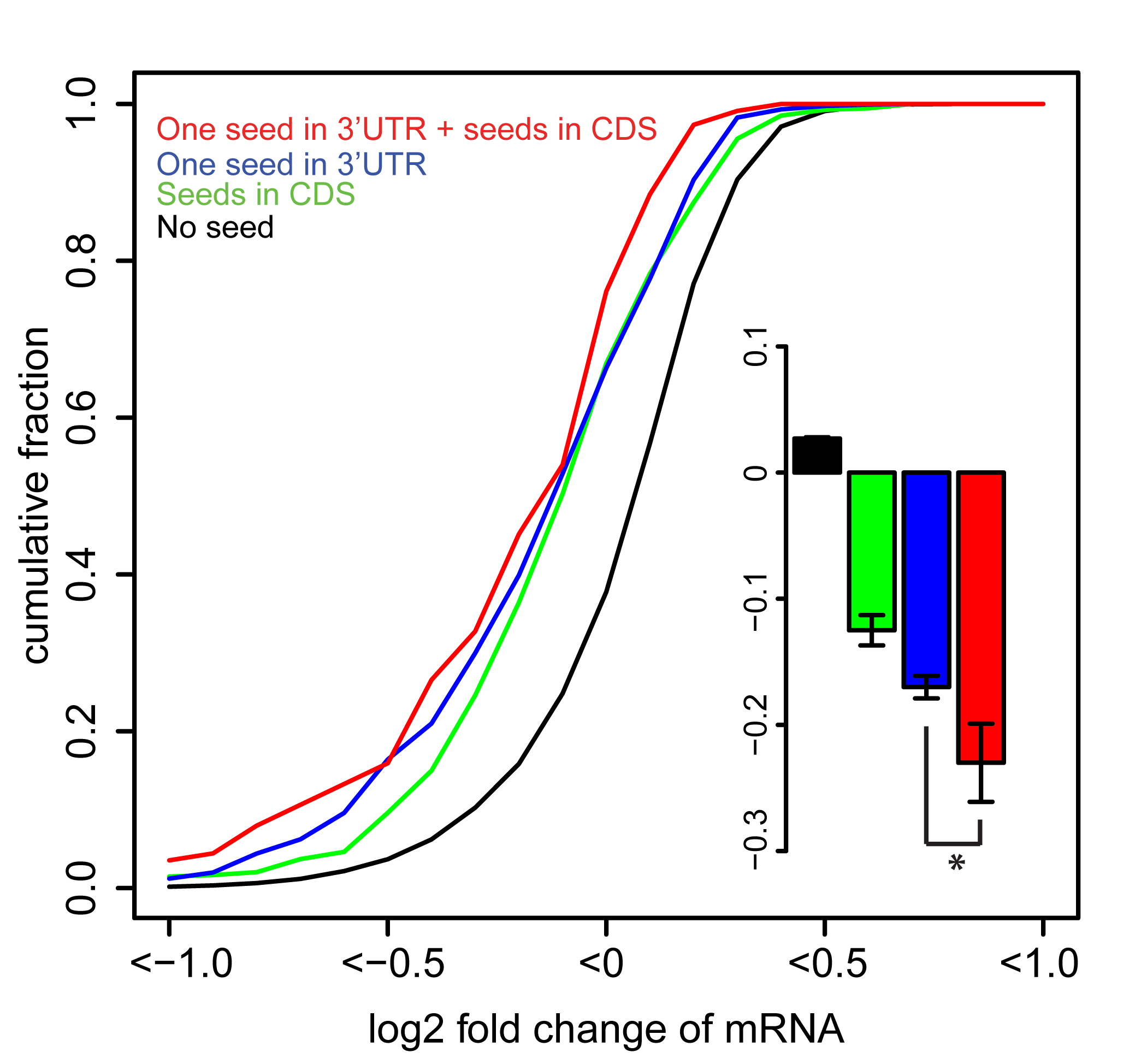

Supplement: Figure S1 — Effect of miRNA target sites in coding regions in AGO2 PAR-CLIP experiment. The figure shows cumulative distributions of log2 fold changes of mRNAs containing one AGO2 binding site in 3′UTRs and additional binding sites in coding regions, one binding site in 3′UTRs, binding sites only in coding regions and no binding site, respectively. The insert shows the mean log2 fold changes (with standard error) of corresponding mRNA groups. *P-value is 0.03 by Wilcoxon test and 0.01 by Kolmogorov-Smirnov test. (TIF) [file pone.0018067.s001.tif]

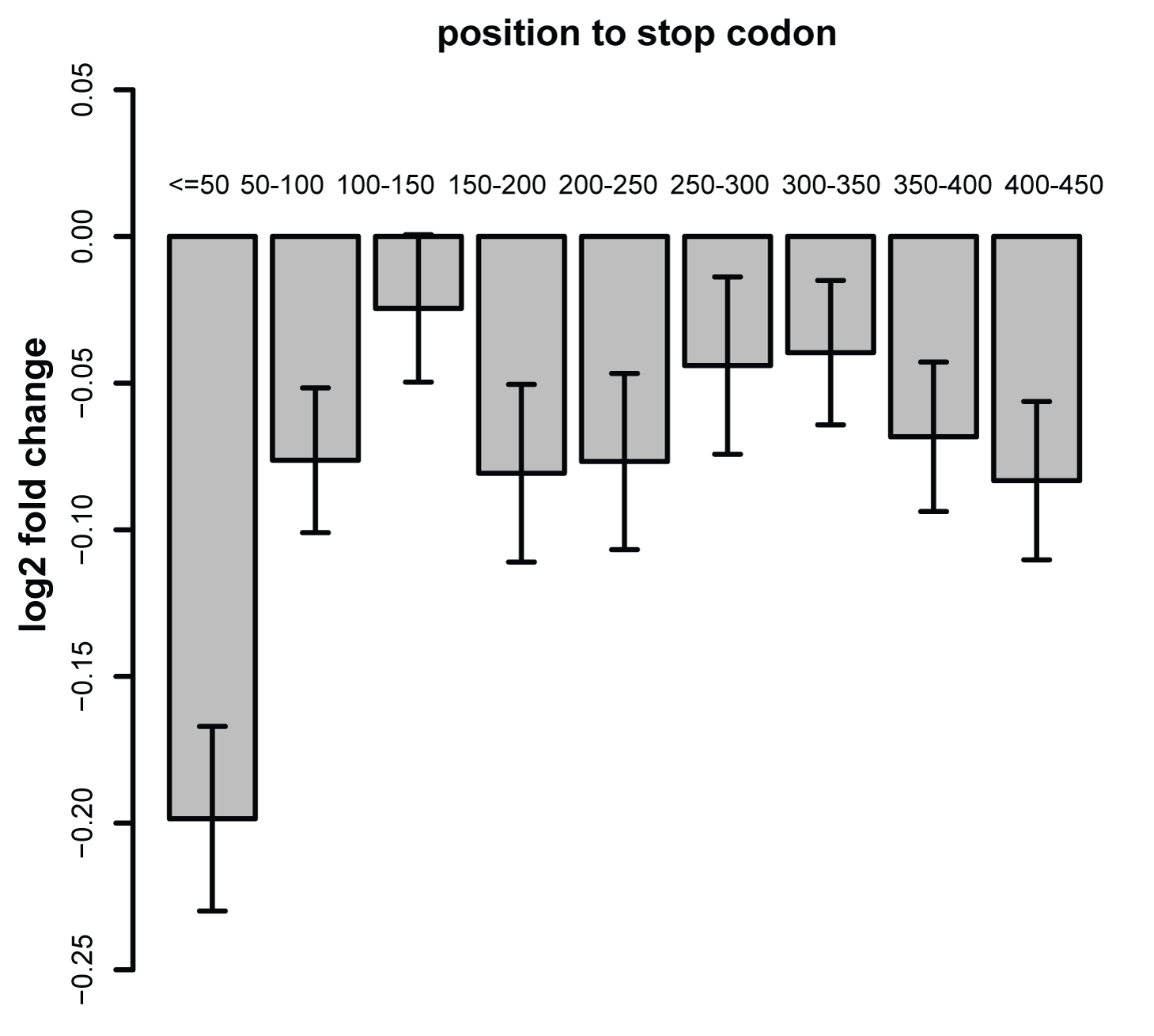

Supplement: Figure S2 — Strong synergistic effect of miRNA seeds in coding region within ∼50 nt of stop codon. mRNAs with 1 3′UTR seed and 1 seed in coding region were grouped according to the distances of the seed in coding region to the stop codon. Averaged log2 fold changes with standard errors were shown. (TIF) [file pone.0018067.s002.tif]
